# Regional anesthesia in the intensive care unit: a single center’s experience and a narrative literature review

**DOI:** 10.1007/s44250-023-00018-w

**Published:** 2023-01-23

**Authors:** Emanuele Russo, Marina Latta, Domenico Pietro Santonastaso, Daniele Bellantonio, Alessio Cittadini, Dario Pietrantozzi, Alessandro Circelli, Emiliano Gamberini, Costanza Martino, Martina Spiga, Vanni Agnoletti, Cesena Intensive Care Unit team, Ferdinando Avolio, Beatrice Benini, Marco Benni, Carlo Bergamini, Giovanni Bini, Luca Bissoni, Giuliano Bolondi, Deborah Campagna, Francesco Cocciolo, Cristian Dell’amore, Benedetta De Santis, Vinicio Dima, Emmanuel Gasperoni, Tommaso Greco, Luca Gobbi, Diego Marandola, Costantino Mastronardi, Manlio Cosimo Claudio Meca, Luca Mezzatesta, Giampaolo Orsolini, Maria Andrea Palazzo, Silvia Passero, Mario Piccinno, Erika Pirini, Chiara Rosato, Giuseppe Sabia, Flavia Savelli, Giovanni Scognamiglio, Andrea Sica, Federica Spina, Claudia Turrini, Alessandra Venditto, Lorenzo Viola, Sofia Vitali, Maria Chiara Zecchini

**Affiliations:** 1 Department of Surgery and Trauma, Anesthesia and Intensive Care Unit, Maurizio Bufalini Hospital, Cesena, Italy; 2 Anesthesia and Intensive Care Department, Alma Mater Studiorum – Università Di Bologna, Bologna, Italy; 3 Department of Surgery, Anesthesia and Intensive Care Unit, Infermi Hospital, Rimini, Italy; 4 Department of Anesthesiology and Acute Care, Umberto I Hospital of Lugo, Ausl Della Romagna, Lugo, Italy

## Introduction

Patients admitted to the intensive care unit (ICU) are vulnerable to severe pain as a result of tissue injury due to serious illnesses, inflammation, major surgeries, body positioning, medical devices, and traumatic injuries. The management of pain in the critically ill population is emerging as a standard of care in the intensive care unit (ICU).

Although, Schulz-Stübner S. et al. in their work underlined that 95% of physicians and 81% of nurses believed the patient had adequate analgesia [1], it is estimated that as many as 70% of patients experience moderate-to-severe postoperative pain during their stay in the ICU [2]. In particular, it is present in up to 50% of patients at rest and in up to 80% of patients receiving routine care procedures, such as tracheal aspiration or drain removal [3].

Despite being a frequent symptom in the ICU and despite numerous improvements, pain is underdiagnosed, especially in sedated and intubated patients. Inconsistencies in pain assessment result, therefore, in suboptimal pain management [4].

Inadequate pain control can lead to detrimental effects on several organ systems in critically ill patients; effects on the respiratory system such as hypoventilation, decreased ability to cough, atelectasis, pneumonia, or on the cardiovascular system such as tachycardia, or increased myocardial oxygen demand [5].

The consequences of inadequate pain control can include sleep deprivation or agitation. Pain assessment in the critically ill patient may be impaired by sedation because the administration of sedatives may mask some manifestations of pain, and the excessive use of some analgesics may result in over-sedation. So, it is essential to discriminate between the two components, especially adopting a careful assessment of pain during the execution of “sedation holidays,” with an eventual preference for multimodal strategies [6].

Furthermore, pain control in surgical and trauma patients is a major issue. In these patients, a valid chest wall expansion, effective coughing, mobilization, and positioning are the conditions for a rapid weaning from mechanical ventilation, and they are all dependent on adequate analgesia [7].

In postoperative patients, suboptimal pain control can also cause prolonged ileus, nausea, and vomiting, with a major risk of aspiration, then a delay in the healing of wounds, and the risk of infection with increased cytokine production [8, 9].

Recent reports have also suggested that, over the long term, a significant number of patients with pain and discomfort during mechanical ventilation are at higher risk of developing post-traumatic stress disorder [10, 11].

Adequate postoperative and post-trauma pain management is crucial for the achievement of effective rehabilitation. All of these potentially modifiable, destructive sequelae of surgery and injuries can severely limit a patient’s return to a normal life.

Therefore, it is vital that physicians and nurses closely caring for these issues, and adopt an optimal pain management strategy that includes a reduction in noxious stimuli, adequate analgesia, and the promotion of education regarding sedation and analgesia to the ICU staff.

For decades, opioids have been the pillar of acute pain management. Historically, opioids (specifically morphine) were the gold standard for managing moderate to severe pain [12].

Opioid-related adverse drug events are common in hospitalized patients. The most frequent are nausea and vomiting (> 55%), and pruritus (> 33%) [13].

Even short-term use of potent opioid compounds for acute pain can produce clinically significant hyperalgesia [14].

A recent study by Chu et al. [15] suggested that opioid tolerance appears within 1 month of initiating therapy with oral morphine in patients with chronic pain. As the overall exposure to opioids increases, the patient’s risk of persistent opioid use after discharge increases. It is a vicious cycle that results in reliance upon ever-increasing amounts of opioids for relatively lower acute and/or chronic pain relief.

Aside from the numerous and common side effects associated with opioids, the latest evidence details the possibility of an increased risk of metastasis associated with opioids use during anesthesia, although there is no uniformity on this topic in the current literature [16, 17]. In particular, the immunomodulating role of opioids on the immune system is not fully understood, and it may have relevant implications in critically ill patients [18], as well as long-term effects of the onset of tolerance [19].

Opioid-free anesthesia can improve post-operative outcomes in several surgical settings without evidence of adverse effects on patient safety and pain management [20].

Evidence has demonstrated that multimodal analgesia can enhance pain relief, and results in fewer adverse reactions than therapy provided through use of a single medication or modality (monomodal analgesia) [21].

As part of efforts to facilitate a patient’s return to normal function, enhanced recovery after surgery (ERAS) protocols have been widely implemented, first for elective colorectal surgery and now for other types of surgery, including emergency laparotomy. Two major components of ERAS pathways include setting of patient expectations prior to surgery and opioid minimization through use of multimodal and regional anesthetia strategies [22].

Extrapolating from lessons learned from enhanced recovery after surgery protocols, surgical intensivists are increasingly utilizing multimodal pain regimens (MMPRs) in critically ill surgical patients recovering from major surgical procedures and injuries [23].

Multimodal analgesia allows for a decrease in opioid use when combined with nonopioids in relieving pain [24].

This strategy ensures a more focused and patient-centered plan of care.

Based upon this premise and numerous evidence-based clinical trials, the American Pain Society (APS) and the American Society of Anesthesiologists (ASA) pointed out multimodal analgesia for pain management [25].

The time of opioids as the pillar of acute pain management in the surgical ICU has begun to be supplemented by other options.

Locoregional ultrasound-guided anaesthesia procedures enhanced the reliability of nerve block practice. Such improvements are also extremely useful as an aid or alternative to general anaesthesia in frail patients with reduced cardiorespiratory reserve or those taking chronic therapies with impact on haemostasis [26]. 

Ultrasound-guided interfascial blocks of the thoracic and abdominal wall are safe and relatively easy to perform [27].

For pain treatment in thoracic surgery and in thoracic trauma, the blocks more commonly used are the thoracic paravertebral (TPV) block and the erector spine plane (ESP) block, in addition to thoracic epidural analgesia [28].

Since the 1980s, the interest in, and impact of, thoracic paravertebral blocks in reducing post-operative pain for several thoracic surgical procedures have grown. By incorporating enhanced recovery after surgery (ERAS) protocols into their clinical practices, there has been a reduction in narcotic drugs, and also an improvement in complication rates and a decrease in length of stay [29]. Nowadays, it is often used as an adjunct to multimodal post-operative analgesia in thoracic surgery [30], breast surgery [31], renal surgery, video-assisted thoracoscopic surgery, and minimally invasive cardiac surgery.

As concerns abdominal surgery, epidural analgesia has long been recognized as the gold-standard technique for analgesia after abdominal surgery [32]. However, growing evidence supports the effectiveness of regional blocks, such as the erector spinae plane (ESP) block and the transversus abdominis plane (TAP) block. Although both are good ways to relieve postoperative pain after abdominal surgery, a recent meta-analysis states that ESP block does not provide better clinical analgesia than the TAP block [33]. In addition, TAP block appears to provide both an effective analgesia and a significant reduction in opioid use on the first postoperative day after colorectal surgery [34].

From operating rooms, peripheral blocks have begun to spread in the ICUs both for pain control in the post-surgical setting as well as in patients with several conditions.

In 2020–2021 the high risk of exposure associated with airway instrumentation during SARS-Cov2 infection led to increased focus on regional anesthesia as an alternative to intubation and general anesthesia. Several papers emphasized that ultrasound-guided regional blocks are safer for medical personnel and patients in the era of Covid 19 [35].

In the past 20 years, in accordance with the growing interest and widespread diffusion of LRA in the critical patient, selected reviews have explored the topic [6, 36].

Ongoing and continuing focus on the topic has led researchers to investigate the possible role of LRA and multimodal analgesia in the neurocritical patient as well [37].

Therefore, this case study aimed to evaluate the improvement of pain management in our intensive care unit considering the change in organization and the increasing contribution of anesthesiological skills.

## Case study

We retrospectively analyzed anonymized and aggregated data on all in-patients admitted to the ICUs of Maurizio Bufalini Hospital at Cesena, from January 1 2015 to December 31 2021.

The types of loco-regional anesthesia we considered in this study are those practiced in our intensive care unit. Those include a neuraxial approach (meaning epidural analgesia), peripheral nerve blocks, and nerve blocks with a perineural catheter.

Our study population is, therefore, represented by all patients during their ICU stay. We searched for patients who had received one of these types of analgesia, and we analyzed data on pathology at admission date (like trauma and surgery—both elective and emergency), on the type of treatment given (peridural catheter placement, peripheral nerve block administration, perineural catheter placement).

The ICU where the analysis was performed serves as a Hub center for trauma, neurosurgical, and neuroradiological conditions. The number of beds historically was 18, which increased to 23 from March 2020 [38, 39].

In the years prior to the study period, the study hospital had two different intensive care units staffed by two different departments. The merger of the two departments was started in 2014, but the medical staff of the two intensive care units continued largely to be distinct. The head of the Anesthesia and Intensive Care Department changed in March 2016, and gradually the teams (including resuscitative, anesthesiological, neurocritical care, and trauma) moved toward integration, expanding the number of physicians with expertise in different subspecialties. Specifically, since 2017, physicians with distinctive anesthesiologist skills have started to shift in the ICU. The unifying process was fully completed during 2019.

**Fig. 1:**
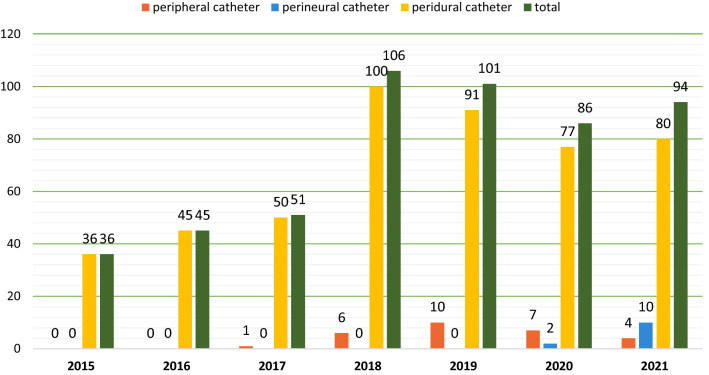
Number and subtype of LRA procedures in ICU from 2015 to 2021

As can be observed in Fig. 1, the trends of every type of loco-regional anesthesia are increasing sharply.

The total number of patients who received at least one loco-regional analgesia treatment increased from 36 in 2015 to 94 in 2021, with a maximum of 106 in 2018.

The first peripheral nerve block was performed in 2017, and the first perineural catheter was placed in 2020; the annual number of these procedures quickly rose to 10 in 2021.

The number of admissions and the case mix of surgical and trauma patients did not increase over the observed period to justify the increase in the application of loco-regional anesthesia techniques (see Table 1). Indeed, in 2020–21, the number of patients eligible for LRA decreased slightly due to the impact of the SARS-Cov-2 pandemic.

**Table 1:** Total number of patients admitted to ICU from 2015 to 2021, split by year and by different subgroups

	2015	2016	2017	2018	2019	2020	2021
No. of patients admitted in intensive care unit	753	761	816	885	944	987	1056
% Surgical patients (emergency and elective)	57.8	61.5	56	55.3	54.2	54.4	51.5
% Trauma patients	31.5	38.5	29.8	34	31	22.9	25.8

## Discussion

In a multicenter study conducted between August 2011 and May 2012, Jabudensen et al. reported the utilization of epidural anesthesia in 6.38 percent of patients admitted to the ICU [40].

In the ICU under study, the percentage of patients submitted to locoregional analgesia techniques rose from 4.78 in 2015 to 8.90 in 2021. Furthermore, two completely new procedures for the setting were introduced, namely the peripheral nerve block and the perineural catheter.

We found a remarkable increase in the use of loco-regional anesthesia techniques during the period 2015–2021. The absolute numbers may seem low, but it is the trend that is important.

Comparing different case mixes can be misleading. Even knowing some characteristics of the population, for example, not all trauma and post-surgical patients can benefit from LRA techniques. However, it seems possible that LRA techniques were slightly underutilized before the integration of the different intensivist and anesthesiologist teams.

The increasing use of loco-regional anesthesia techniques in our ICU is likely due to both the world-wide spreading of the echo-guided approach and the changes in team composition.

Boosting the skills of the intensivist team certainly helped to gradually undermine the paradigm by which ICU analgesia should coincide solely with intravenous opioid analgesia.

Obviously, our experience does not answer the complex issues of the suitability or otherwise of having hyper-specialized teams. However, human resource governance and the impact of different organizing models on the volume of services provided, and health outcomes, is an absolute priority for healthcare management.

Assessing the impact on clinical outcomes of loco-regional analgesia techniques and quantifying the relationships between the resources employed and the volume of health care benefits delivered is not an aim of this case study.

Such aims would require well-designed studies with sizable amounts of data to explore; this paper is also meant to be a call for research on these issues.

Nonetheless, we would highlight that having progressively integrated the different teams has contributed to the spread of loco-regional analgesia techniques in the ICU.

Furthermore, it should be kept in mind that in Italy the course of study provides the same postgraduate medical school specialization for intensive care and for anesthesia [41].

In conclusion, we believe that focus on pain care deserves a pivotal role in the treatment of critically ill patients, and that pain management should be recorded in the medical record, and should lead to pain-control strategies. The steady increase in attention to pain issues, in addition to being crucial from a pathophysiological and clinical point of view, is part of an overall process of humanizing intensive care.

## Declarations

### Data availability

The datasets generated during and/or analysed during the current study are available from the corresponding author on reasonable request.
